# Life history traits and phenotypic selection among sunflower crop–wild hybrids and their wild counterpart: implications for crop allele introgression

**DOI:** 10.1111/eva.12261

**Published:** 2015-05-12

**Authors:** Matthew A Kost, Helen M Alexander, D Jason Emry, Kristin L Mercer

**Affiliations:** 1 Department of Horticulture and Crop Science, The Ohio State UniversityWooster, OH, USA; 2 Department of Ecology and Evolutionary Biology, University of KansasLawrence, KS, USA; 3 Department of Biology, Washburn UniversityTopeka, KS, USA; 4 Department of Horticulture and Crop Science, The Ohio State UniversityColumbus, OH, USA

**Keywords:** demographic swamping, early season traits, genetic assimilation, *Helianthus annuus*, hybrid zone evolution, introgression, life history traits, phenotypic selection

## Abstract

Hybridization produces strong evolutionary forces. In hybrid zones, selection can differentially occur on traits and selection intensities may differ among hybrid generations. Understanding these dynamics in crop–wild hybrid zones can clarify crop-like traits likely to introgress into wild populations and the particular hybrid generations through which introgression proceeds. In a field experiment with four crop–wild hybrid *Helianthus annuus* (sunflower) cross types, we measured growth and life history traits and performed phenotypic selection analysis on early season traits to ascertain the likelihood, and routes, of crop allele introgression into wild sunflower populations. All cross types overwintered, emerged in the spring, and survived until flowering, indicating no early life history barriers to crop allele introgression. While selection indirectly favored earlier seedling emergence and taller early season seedlings, direct selection only favored greater early season leaf length. Further, there was cross type variation in the intensity of selection operating on leaf length. Thus, introgression of multiple early season crop-like traits, due to direct selection for greater early season leaf length, should not be impeded by any cross type and may proceed at different rates among generations. In sum, alleles underlying early season sunflower crop-like traits are likely to introgress into wild sunflower populations.

## Introduction

The merging of crop and wild habitats on the edge of agricultural fields (i.e., ‘hybridization of the habitat’; Anderson 1948) can produce ecological niches favorable to crop–wild hybrids possessing new ‘genetic systems of adaptation’ (Anderson and Stebbins 1954, pp. 378). This can lead to hybrid zones containing crop–wild F_1_ hybrids, advanced generation hybrids, and their wild counterpart (Anderson 1949). If F_1_ hybrids are fertile and backcross onto either parent population (Anderson and Hubricht 1938), or if they sib-cross (Heiser 1973), introgression of alleles from one population to another can ensue as long as natural selection favors them or genetic regions linked to them (Stebbins 1959). As gene flow and subsequent introgression between crop and wild populations can be asymmetric toward wild populations (Ellstrand and Elam 1993; Papa and Gepts 2004; Papa 2005), many researchers have investigated possible introgression of crop alleles into wild populations in cases where crop and wild types are sympatric, are able to ucodep>type/ucodep>pollinate, and are interfertile.

In situations where crop–wild hybridization and subsequent introgression into wild relatives of crop plants does occur, there can be a number of consequences (Ellstrand et al. 1999; Ellstrand 2003a; Haygood et al. 2003). Many have focused on the possibility of crop toward wild gene flow increasing the invasiveness of wild populations (Snow and Moran-Palma 1997; Ellstrand and Schierenbeck 2000; Hooftman et al. 2005). Another equally important concern is genetic diversity loss through the processes of demographic swamping and genetic assimilation (Levin et al. 1996; Wolf et al. 2002; Haygood et al. 2003). If gene flow into a wild population produces hybrids with reduced fitness when compared to the parental populations (i.e., outbreeding depression), then lack of self-replacement may lead to demographic swamping (Ellstrand 1992; Wolf et al. 2002; Haygood et al. 2003). Genetic assimilation, or the replacement of wild alleles with cultivated ones (Ellstrand 1992; Papa et al. 2005), is likely when hybrids experience minimal or no reduction in fitness (Wolf et al. 2002), or when asymmetric introgression into wild populations occurs due to numerical superiority of crop plants (Brock 2004). Cultivated varieties often possess less genetic diversity than wild populations; therefore, genetic assimilation can decrease genetic diversity of wild populations at genetic loci under selection and in linked regions (Fisher 1930; Falconer and Mackay 1996). These potential consequences warrant continued study on the process of crop–wild hybridization and subsequent introgression of specific traits and/or suites of traits.

*Helianthus annuus* (sunflower) is an ideal system for studies of these topics. Abundant research demonstrates that gene flow and introgression between cultivated *H. annuus* (cultivated/crop sunflower) and wild *H. annuus* (common sunflower; hereafter wild sunflower) occurs. Cultivated and wild sunflower overlap in flowering time throughout the range of sunflower cultivation; the two share insect pollinators; and they readily hybridize even at distances up to, and likely exceeding, 1000 m (Arias and Rieseberg 1994; Linder et al. 1998; Burke et al. 2002a). Alleles from cultivated sunflower populations have also been shown to readily introgress into wild populations and remain for multiple generations (Whitton et al. 1997; Linder et al. 1998). Even though many crop traits may reduce fitness under wild conditions, several studies have demonstrated that transgenes and traditionally bred traits providing fitness benefits in wild populations should introgress (Massinga et al. 2003; Snow et al. 2003; Presotto et al. 2012; but see Burke and Rieseberg 2003). While these studies show that cultivated alleles maintain the potential to introgress into wild sunflower populations, they do not provide insight into how selection operates to introgress particular traits and their underlying alleles.

Differences between sunflower crop–wild hybrids and their wild counterpart for life history traits and fitness play a role in determining how introgression of cultivated alleles proceeds within wild populations. As demonstrated in sunflower, F_1_ hybrids and their wild counterpart can differ in growth, phenology, and life history traits, such as probability of germinating, seedling size, survival to flowering, flowering time, seed size, and fecundity (Snow et al. 1998; Alexander et al. 2001; Mercer et al. 2006a; Mercer et al. 2007). In the field, F_1_ sunflower crop–wild hybrids produced on wild maternal plants overwinter and germinate in high proportions during the spring and may be more likely to survive until reproduction than wild plants (Snow et al. 1998; Mercer et al. 2006b). Although the process of introgression does not occur until the F_2_ or backcross generations (Anderson and Hubricht 1938; Rieseberg and Wendel 1993), these findings suggest life history characteristics of the F_1_ generation do not provide a strong barrier to the introgression of cultivated sunflower alleles into wild populations. Determining which advanced generation hybrid cross types also facilitate introgression can aid in the identification of potential cross type genetic route(s) of introgression following sunflower crop–wild hybridization. For instance, if a certain crop–wild cross type does not overwinter and survive until flowering, then that cross type will not contribute to the process of introgression. While measuring life history traits can provide much insight into the process of introgression, understanding how natural selection occurs in hybrid zones and on different hybrid zone cross types is necessary to determine: (i) crop-like traits likely to introgress into wild populations and (ii) routes through which introgression of these traits will likely proceed.

Phenotypic selection analysis can be used to gain understanding of how natural selection operates within hybrid zones. Such understanding can in turn provide insight into how crop alleles controlling trait values favored by natural selection may introgress into wild populations. Revolutionary work by Pearson (1903) clarified that multivariate statistics could differentiate direct from indirect selection. This was expanded and generalized by others (Lande and Arnold 1983; Arnold and Wade 1984), providing a framework for exploring adaptive evolution in wild populations. Phenotypic selection, the association between fitness and quantitative phenotypic variation among individuals, also estimates the direction and strength of selection occurring on correlated traits (Lande and Arnold 1983). Mercer et al. (2011) performed phenotypic selection analysis on wild sunflower maternal families to ask whether selection varied across genetic families when grown together as a single population. They found that both the strength and direction of selection on particular traits differed across families. Here we employ a similar analysis on four sunflower cross types grown and interacting together as a single population, under field conditions, to determine whether natural selection affects these genetic groupings differently. The four cross types—three sunflower crop–wild hybrids (BC_w_, F_1_, and F_2_) and their wild counterpart (W)—all can be expected to co-occur in natural sunflower crop–wild hybrid zones. The information gleaned from cross type specific phenotypic selection analyses can clarify routes by (i.e., cross types through) which introgression of alleles underlying crop-like traits, and genetic regions linked to these alleles, is likely to proceed by elucidating selection intensity differences among cross types.

During this study, we quantified differences in growth, phenology, and life history traits, as well as survival between BC_w_, F_1_, and F_2_ sunflower crop–wild hybrid cross types and their wild counterpart in an experimental crop–wild hybrid zone field setting to identify potential barriers to the process of introgression. Our focus was on early season traits, from seedling emergence to anthesis; we planted seeds in the fall so all cross types could experience natural overwintering. We then employed phenotypic selection analysis to clarify selection dynamics operating within crop–wild hybrid zones and to identify early season crop-like traits likely to introgress into wild populations. By determining whether natural selection differentially occurred on the various cross types, we assessed the likely cross types through which introgression of the traits under study may proceed and the likelihood of it doing so (i.e., based on the size of selection intensities). We focused on three overarching questions: (i) To what degree are cross types capable of overwintering and emerging in the spring and do they differ in emergence time? (ii) Do differences exist between cross types for growth traits and survival to anthesis? (iii) To what degree are early season traits related to survival to anthesis and are cross types differentially affected by natural selection? Overall, we combined the study of growth, phenology, life history traits, and phenotypic selection analyses to elucidate crop toward wild introgression of early season traits following sunflower crop–wild hybridization. Finally, we employed our findings to shed light on the likelihood of posthybridization wild sunflower genetic diversity loss through the processes of demographic swamping and genetic assimilation.

## Materials and methods

### Study system

Cultivated sunflower was domesticated from wild sunflower in the eastern United States more than 4000 years ago (Crites 1993; Harter et al. 2004; Blackman et al. 2011). While cultivated and wild sunflower are cross-compatible due to their common ancestry, domestication and continued selection has led to their differentiation in many morphological and fitness related traits (reviewed in Burke et al. [Bibr b14][Bibr b15]).

### Plant material

Sunflower achenes (hereafter seeds) were generated by hand pollination in 2009 at Waterman Farm in Columbus, Ohio, resulting in four cross types: wild (wild × wild), BC_w_ (wild × F_1_), F_1_ (wild × crop), and F_2_ (F_1_ × F_1_). The F_2_ cross type was produced on F_1_ maternal plants, while all other cross types were produced on wild maternal plants. Cross types produced on wild maternal plants differ in the mean percent crop alleles they possess: wild, 0%; BC_w_, 25%; and F_1_, 50%. As both the F_1_ and F_2_ cross types possess 50% mean percent crop alleles, differences observed between these two cross types may be attributed to: (i) being produced on different maternal plants (maternal effects), (ii) epistasis, (iii) recombination, (iv) the uncovering of recessive alleles, or (v) overdominance (Rieseberg et al. 1999). The wild sunflower germplasm was a bulk of 10 populations, collected from multiple locations (agricultural fields, construction sites, wetlands, and roadsides), within 30 km of our main field experiment in Lawrence, Kansas. In 2007, we generated F_1_ seed by crossing USDA inbred line HA89 pollen onto 20 wild maternal plants derived from the Kansas collections. In 2009, wild plants from the Kansas germplasm collections, F_1_ plants from the 2007 crosses, and HA89 cultivated plants were grown in separate blocks within 40 m of one another in a uniform experiment station field with optimal nutrients for seed development. We performed hand pollinations on wild maternal plants using wild, F_1_, or crop pollen to generate the wild, BC_w_, and F_1_ cross types, respectively. We generated F_2_ seed by performing hand pollinations between the F_1_ plants. We selected 18 wild and 18 F_1_ maternal plants and considered the seeds produced on each, for a given cross type, to be a maternal family. As such, all cross types produced on wild plants were from the same 18 plants, while all F_2_ families came from a distinct 18 plants. Multiple pollen donors were employed during crossing to increase the amount of diversity present in a given family and cross type.

### Field experimental design

We conducted the field experiment in a 5.4 hectare brome field at the University of Kansas Field Station in Jefferson County, Kansas. As the location is within the native range of wild *H. annuus*, the area to be planted was rototilled in the spring of 2009 and allowed to go fallow to ensure the absence of sunflower seeds in the seed bank; no sunflower seedlings were observed. The experimental site was rototilled again before the planting in November 2009. We employed the method outlined in Mercer et al. (2011) of using Gorilla Glue (The Gorilla Glue Company, Cincinnati, OH, USA) to affix seeds to labeled swizzle sticks prior to planting to allow us to follow particular genotypes that overwinter naturally. Preliminary analysis of a side experiment showed no effect of swizzle sticks or Gorilla Glue on germination (data not shown).

Our study was performed in the context of a factorial competition experiment. Here we present a full experimental design, but emphasize that the focus of this study was not on the competition treatments per se (given the absence of cross type by treatment interactions; see Results), but instead on life history traits of sunflower crop–wild hybrid zone cross types and the differences in intensities of phenotypic selection occurring on these cross types. Specifically, our field study was arranged as a split-plot design with five treatment factors and six blocks. Each of 12 main plots in each block was randomly assigned a factorial combination of three competition factors, and the 72 subplots in each main plot were randomly assigned sunflower cross type and maternal family combinations. The factors combined to make up the competition treatments consisted of two manipulations of intraspecific competition—density of sunflower seeds (low, 100 seeds/m^2^; medium, 255 seeds/m^2^; high, 495 seeds/m^2^) and frequency of hybrids (15 and 40%)—and also a manipulation of interspecific competition (vegetation intact or removed). We determined appropriate planting densities using wild population surveys performed in 2009 and expected wild sunflower emergence percentages from the literature (as in Mercer et al. 2011). Each block consisted of two 14.3 m × 1.35 m strips. In each main plot, we sowed 18 focal seeds (one per family) from each of the four cross types except in the low density 15% hybrid plots, where we removed a portion of the hybrid focal seeds to maintain the appropriate percent hybrids within the plot. We then applied matrix seed as described in Mercer et al. (2011) until we achieved the correct densities and hybrid percentage for each main plot. After planting, there were a total of 4824 focal seeds in the experiment.

### Data collection

We evaluated emergence on all focal plants and collected data on early season growth and survival to anthesis on all focal plants that emerged. Beginning in mid-March 2010, we collected data on emergence three times a week until mid-May and once a week until emergence all but ceased on 25 May. We marked all emerged focal seedlings, a total of 2670. Seedlings that emerged >1.5 cm away from a focal swizzle stick or focal sticks having two seedlings in close proximity were not considered focal plants. On 26 April (early season; census 1), 17 May (census 2), and 28 June (census 3), we recorded plant height (base to apical meristem), length of longest leaf (petiole to tip; hereafter leaf length), width of longest leaf (distance at thickest portion of leaf; hereafter leaf width), number of nodes, and number of petiole leaves. We recorded date of first flower twice a week beginning on 1 July and ending on 4 September. Whether or not a plant flowered (i.e., survival to anthesis) was then used to approximate reproductive success of emerged seedlings from the four cross types. We also calculated a related metric—probability that a planted seed flowered in the first year—using all seed planted in the fall. This final metric is equivalent to multiplying % spring emergence by survival to anthesis, and therefore, it provides a more complete view of the sunflower life cycle.

### Data analysis

We executed statistical analysis in SAS version 9.3 (SAS Institute, Cary, NC, USA). Proc Glimmix was used for analysis unless otherwise stated.

Percent spring emergence and probability that a planted seed flowered in the first year were calculated from the 4805 original focal seeds planted in the fall. Analysis on the remaining response variables (emergence date, plant height, leaf length, leaf width, number of nodes, number of petiole leaves, and survival to anthesis) was performed on all marked (i.e., emerged) plants that remained from the 2670 plants that emerged in the spring. First, we calculated correlations among traits with Proc Corr to identify a representative and moderately independent set of traits. Because correlation coefficients among all traits were significant at *P* < 0.001 and ranged from 0.6125 to 0.955 (Table S1), we report a represented and moderately independent subset of traits (Lande and Arnold 1983): emergence percentage and timing, leaf length, and height.

Second, we performed anova using mixed models—where the fixed effects included density, percent hybrid, interspecific competition, and cross type, as well as all interactions among these factors and random effects included block and interactions with block. As the aim of this study was to elucidate differences in emergence and life history traits between cross types and to relate any observed differences to survival to anthesis, we did not include family as a factor in our analyses. Due to the split-plot nature of our design, we used two separate error terms. Our main plot error term included interactions between block, percent hybrid, interspecific competition, and density of seeds; our subplot error term also included interactions of these same factors with cross type. Least squared means were generated for all traits and Tukey–Kramer adjustments were used for mean separations. To follow up on anova results for emergence date, we performed failure-time analysis using Proc Lifetest and compared seedling emergence curves for each cross type using a log rank test.

Third, we performed phenotypic selection analysis by employing logistic regressions to estimate selection coefficients (i.e., selection differentials and gradients) for our binary fitness variable—survival to anthesis (Janzen and Stern 1998). Selection differentials represent the combination of direct and indirect selection, while selection gradients represent direct selection. Comparing the two, therefore, provides a way to determine how selection on one trait is influenced by selection on other traits (Lande and Arnold 1983). We predicted survival to anthesis from each of three continuous census one traits (early season height, early season leaf length, and emergence date) while accounting for variation introduced by all experimental factors mentioned in the anova model above. To estimate overall selection differentials (*s*), each of the early season response variables were included in the model individually. To estimate overall selection gradients (*β*), all three early season response variables (emergence, height, and leaf length) were included in a single model. As we also had interest in determining cross type specific selection differentials and gradients, we generated models that included either a particular early season response variable and its interaction with cross type (*s*) or all three early season response variables and their interaction with cross type (*β*). In addition, we investigated density-specific selection differentials and gradients using similar models as density was the only factor besides cross type that influenced both an early season trait (i.e., early season height, early season leaf length; Tables S2 and S3) and, importantly, survival to anthesis (Table S4). For pairwise comparisons among cross types or densities of selection coefficients for the same trait, we used a Holm–Bonferroni step-down adjustment for multiple comparisons (Holm 1979). To ensure all cross types and density-specific selection coefficients were significantly different from zero, we ran a set of models where only the trait by treatment (cross type or density) interaction(s) was included.

Finally, we calculated from these results standardized (*s*’ and *β*’) and transformed (*s-avgdif and β-avggrad*) selection coefficients along with standardized standard errors. The transformed coefficients can be directly used in equations such as the breeder's equation to describe microevolutionary change (Janzen and Stern 1998). Standardized selection differentials and standard errors were generated by dividing selection differentials by trait standard deviations, and standardized selection gradients and standard errors were generated by multiplying selection gradients and standard errors by trait standard deviations (Janzen and Stern 1998; Matsumura et al. 2012). Transformed selection coefficients were generated by: (i) using predicted fitness (W) of individuals to calculate the average of W(1-W), (ii) multiplying the average of W(1-W) by the inverse of relative fitness, and then (iii) multiplying the product by the standardized selection coefficients (*s*’ and *β*’) (for further details, see Janzen and Stern 1998).

## Results

Although our competitive treatments did have individual and combined effects on some of our response variables (Tables S2–S5), given our objectives and the fact that none of the competitive treatments interacted with cross type to affect our response variables, we limit our discussion to cross type effects on traits.

### Emergence

**Figure 1:**
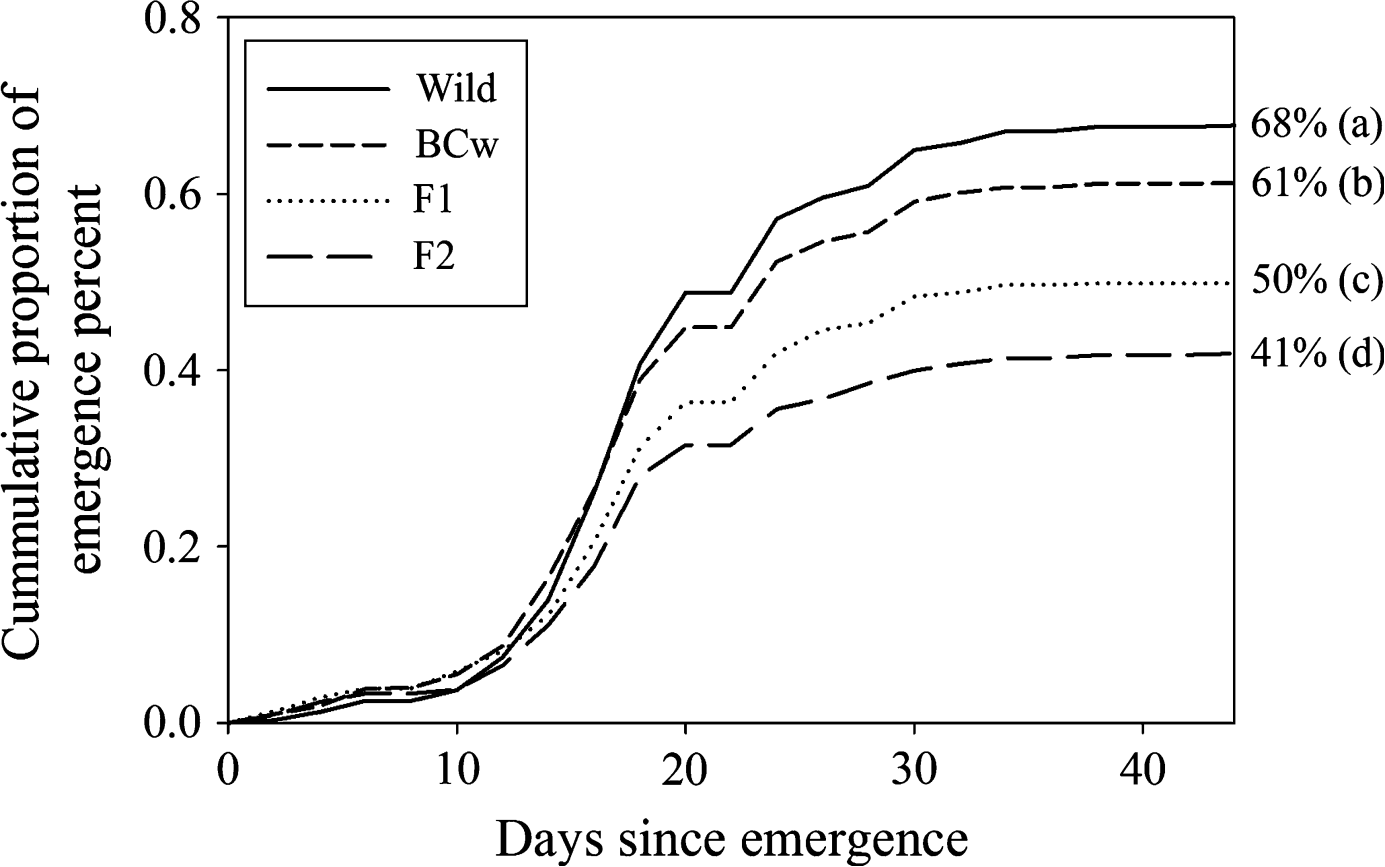
Emergence curves and percent spring emergence for each of the four sunflower crop–wild hybrid cross types grown during our experiment in Jefferson County, Kansas: W, BC_w_, F_1_, and F_2_. Curves were generated using data from all emerged individuals (*n* = 2670), while percent spring emergence was generated using data from all original fall planted focal seeds (*n* = 4805). Spring emergence percentages for each cross type, along with mean separation results for those percentages, are located to the right-hand side of each curve. Standard errors ranged from 0.016 to 0.019. Cross types marked with different letters are significantly different at 0.05 level—Tukey–Kramer adjustment for multiple comparisons. Late emergence events (past day 44) for the W, BC_w_, and F_1_ cross types and standard error bars for the emergence curves have been removed for clarity—standard errors ranged from 0 to 0.0203. The first day of emergence for the experiment was recorded on March 22, 2010 (day 1). Log rank test for failure-time analysis between cross types: *P* = 0.1188. BC_w_–F_1_ backcrossed onto a wild parent.

**Figure 2:**
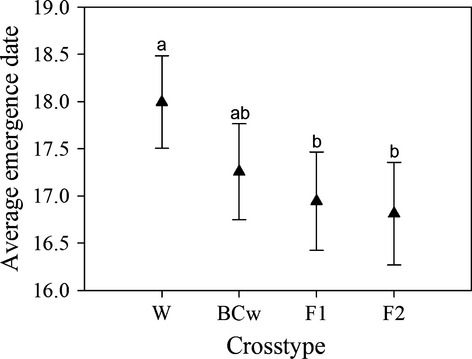
Observed differences in average emergence date for W, BC_w_, F_1_, and F_2_ sunflower crop–wild hybrid cross types originating from our experiment in Jefferson County, Kansas; *n* = 2670. Values are means ± 1 SE. Cross types that are marked with the same letter are not significantly different at the 0.05 level using a Tukey–Kramer adjustment for multiple comparisons.

Focal seedling emergence commenced on 22 March (hereafter day 1) and more than 90% of focal seeds that emerged did so by 4 April. We observed an inverse relationship between percent crop alleles and percent emergence in the three cross types produced on wild maternal plants (*F*_3,180_ = 62.38, *P* < 0.0001; Table S5 and Fig.1); as percent crop alleles increased, emergence percentage decreased (Wild, 68%; BC_w_, 61%; F_1_, 50%). Percent emergence was also influenced by the maternal parent on which seeds were produced. Although both the F_1_ and F_2_ cross types share a population mean of 50% crop alleles, fewer F_2_ than F_1_ cross type seeds emerged (41% vs 50%, respectively; Fig.1). Seedlings of the F_2_ (16.8 days) and F_1_ (16.9 days) cross types emerged earliest, followed by the BC_w_ (17.3 days) and wild (18 days) seedlings (*F*_3,180_ = 4.02, *P* = 0.0084; Table S5 and Fig.2). The greatest difference was between the wild and F_2_ cross types although the observed difference of 1 day may not be biologically significant. Nevertheless, we see a trend of increasing percent crop alleles shortening the average seedling emergence date. Average emergence date was not influenced by the maternal cross type on which seeds were produced, which can be seen by the lack of mean separation between the F_1_ and F_2_ cross types for this trait (Fig.2). The emergence curves for the four cross types do not differ significantly (Log rank test *P* = 0.1188, Fig.1), likely due to the similarity of emergence trends prior to day 15 and despite differences in final emergence percent.

### Vegetative growth

**Figure 3:**
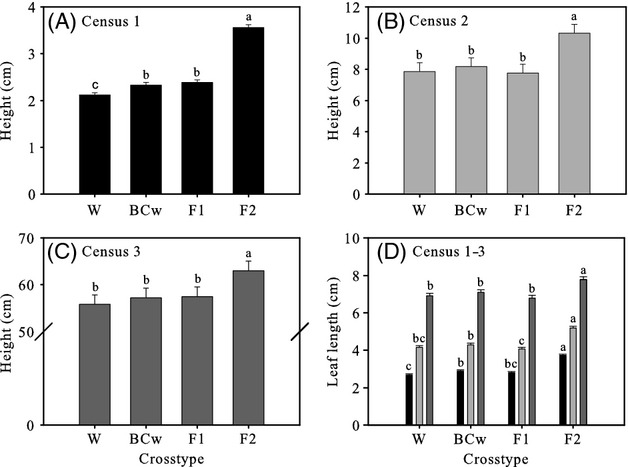
Least squares means for height of W, BC_w_, F_1_, and F_2_ sunflower crop–wild hybrid cross types at (A) census 1 (*n* = 2573), (B) census 2 (*n* = 2562), and (C) census 3 (*n* = 2550). (D) Least squares means for length of longest leaf for W, BC_w_, F_1_, and F_2_ sunflower cross types at census 1 (black bars; *n* = 2511), census 2 (light gray bars; *n* = 2557), and census 3 (dark gray bars; *n* = 2546). Values are means + 1 SE. Cross types that are marked with the same letter are not significantly different at the 0.05 level using a Tukey–Kramer adjustment for multiple comparisons. BC_w_–F_1_ backcrossed onto a wild parent. Note differences in scale on the *y*-axes.

On 26 April (census one), percent crop alleles and maternal parent both affected the height of our cross types. Wild plants were smallest (2.1 cm) followed by the BC_w_ (2.3 cm) and F_1_ (2.4 cm) cross types indicating that increasing height accompanied the presence of crop alleles, although differences were not large (*F*_3,180_ = 242.10, *P* < 0.0001; Table S2 and Fig.3A). Maternal parent significantly influenced height as can be seen from the large difference between the F_2_ (3.6 cm) and F_1_ cross types. On 17 May (census two), we did not see differences among wild-produced cross types—wild, BC_w_, and F_1_ (*F*_3,179_ = 97.30, *P* < 0.0001; Table S2 and Fig.3B); however, the F_2_ cross type remained taller than all wild-produced cross types. On 28 June (census three), we saw a similar pattern of the F_2_ cross type being taller (*F*_3,180_ = 28.07, *P* < 0.0001; Table S2 and Fig.3C).

As was the case with height, cross type affected leaf length (Table S3) with the most salient pattern being that, at all three census dates, leaves were shorter on plants from wild-produced cross types. In particular, the comparisons between the F_1_ and F_2_ cross types were always significant with F_2_s having longer leaves than F_1_s (Fig.3D). Among wild-produced cross types, there was some variation at census one and two (and none by census three), but we did not observe a clear relationship of increasing percent crop alleles increasing leaf length (Fig.3D).

### Survival to anthesis

**Figure 4:**
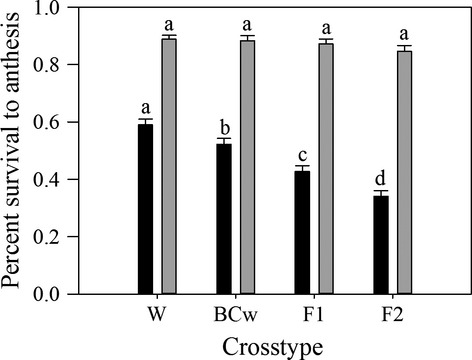
Survival to anthesis for W, BC_w_, F_1_, and F_2_ sunflower crop–wild hybrid cross types from our experiment in Jefferson County, Kansas. Gray bars represent percent survival to anthesis from individuals that emerged in the spring (*n* = 2670). Black bars represent probability that a planted seed flowered in the first year and was calculated from all seed sown in the fall (*n* = 4805). Values are means + 1 SE. Cross-types that are marked with the same letter are not significantly different at the 0.05 level using a Tukey–Kramer adjustment for multiple comparisons.

Sunflower cross type did not have a significant effect on survival to anthesis, which was calculated using only individuals that emerged in the spring (*F*_3,186_ = 1.39, *P* = 0.2462; Table S4); however, the related metric, the probability that a planted seed flowered in the first year, was affected by cross type (*F*_3,180_ = 47.44, *P* < 0.0001; Table S4). When analysis was performed on individuals that emerged in the spring, all cross types had at least 84% survival to anthesis (W = 88.9%, BC_w_ = 88.3%, F_1_ = 87.3%, F_2_ = 84.6%; Fig.4) despite the negligible trend of decreasing survival to anthesis as percent crop alleles increased and a slight reduction in survival to anthesis between the F_2_ as compared to the F_1_ cross type. This vague trend was amplified when we looked at the probability that focal seeds sown in the fall flowered in the first year, such that the greater the percent crop alleles possessed by wild-produced cross types, the lower the survival to anthesis (Wild, 60.4%; BC_w_, 54.3%; F_1_, 43.5%; Fig.4). The means separation between the F_1_ and F_2_ (34.7%) also indicated that seeds produced on F_1_ parents were less likely to flower in the first year than those produced on wild parents.

### Phenotypic selection

#### Overall selection

Both directional selection differentials (*s*) and odds ratios calculated on traits individually, for all cross types combined, indicated an increase in fitness (i.e., survival to flowering) associated with earlier seedling emergence, greater early season height, and greater early season leaf length (Table1). From the odds ratios, the likelihood of within-generation survival was about 4.2 times greater for each 1 cm increase in early season height or leaf length and 1.11 times (1/0.899) greater for each day earlier a seedling emerged (Table1). While selection differentials (*s*) provide information on the observed changes in phenotype due to both direct and indirect selection, selection gradients (*β*) quantify only forces of direct selection occurring on a given trait without influence from indirect selection (Lande and Arnold 1983). Only early season leaf length had a significant selection gradient (*β* = 1.32; Table2), indicating it was the only trait included in the analysis that was under direct selection. The change to nonsignificance of early season height and emergence date when comparing (*s*) and (*β*) further suggested that direct selection on increased early season leaf length likely led to indirect selection on these other traits (Tables1 and 2).

**Table 1:** Overall selection differentials reflecting phenotypic selection patterns on early season height (Es_Ht), early season leaf length (Es_LL), and emergence date (EmergDate)

Selection Differentials—Overall								
	*s*	SE	Odds ratio	95% CI	*s’*	SE(*s’*)	*s*-*avgdif*	Signif.
Es_Ht	1.43	0.11	4.198	3.398–5.187	1.37	0.10	0.13	****
Es_LL	1.42	0.092	4.157	3.471–4.979	1.28	0.08	0.10	****
EmergDate	−0.11	0.0089	0.899	0.884–0.915	−0.015	0.0013	−0.0020	****

Selection differentials (*s*) and their standard errors (SE); odds ratios (Odds ratio) and their 95% confidence interval (95% CI); standardized selection differentials (*s’*) and their standard errors SE(*s’*); and average selection differentials (*s*-*avgdif*) for early season height (Es_Ht), early season leaf length (Es_LL), and emergence date (EmergDate). Significance (Signif.) for a trait's selection differential—trait experienced changes in phenotype due to selection (direct and indirect). Significance: ****P < 0.0001.

**Table 2:** Overall selection gradients reflecting phenotypic selection patterns on early season height (Es_Ht), early season leaf length (Es_LL), and emergence date (EmergDate)

Selection Gradients—Overall								
	*β*	SE	Odds ratio	95% CI	*β*’	SE (*β*’)	*β*-*avggrad*	Signif.
Es_Ht	0.069	0.14	1.071	0.808–1.419	0.072	0.15	0.0056	ns
Es_LL	1.32	0.13	3.738	2.878–4.855	1.47	0.15	0.11	****
EmergDate	−0.15	0.017	0.985	0.954–1.018	−1.01	0.12	−0.079	ns

Selection gradients (*β*) and their standard errors (SE); odds ratios (Odds ratio) and their 95% confidence interval (95% CI); standardized selection gradients (*β*’) and their standard errors SE (*β*’); and average selection gradients (*β*–*avggrad*) for early season height (Es_Ht), early season leaf length (Es_LL), and emergence date (EmergDate). Significance (Signif.) for a trait's selection gradient—trait was a direct target of natural selection. Significance: ****P < 0.0001, ns—not significant, 0.05.

#### Selection by cross type

All cross type specific selection differentials (*s*) were significantly different from zero for all traits indicating each cross type experienced selection on all traits (Table3). In addition, selection differentials (*s*) varied among cross type for these traits as is evidenced by a significant interaction between each trait and cross type (Table3). By contrast, only leaf length experienced direct selection (*β*)—cross types significantly differed from zero (Table4). The significant interaction between leaf length and cross type indicates variation in the intensity of direct selection among cross types occurred on this trait (Table4). Direct selection on leaf length was greater for the wild (1.76) and BC_w_ (1.66) cross types than for the F_2_ (0.66) cross type; the F_1_ (0.87) cross type was nonsignificantly intermediate to these two groupings (Table4). We observed a consistent cross type specific trend of a decrease in direct selection (*β*) on early season leaf length corresponding with reduced total (direct and indirect) phenotypic selection (*s*) occurring on height and emergence date (Table3).

**Table 3:** Cross type specific selection differentials for early season height (Es_Ht), early season leaf length (Es_LL), and emergence date (EmergDate)

Selection Differentials—Cross type									
	*s*	SE	Odds	95% CI	*s’*	SE(*s’*)	*s*-*avgdif*	Signif.	RS
Es_Ht*Cross type									****
W	1.63	0.33	5.084	3.325–7.773	2.07	0.42	0.18	****	ab
BC_w_	1.79	0.33	6.009	3.940–9.166	2.17	0.40	0.24	****	a
F_1_	1.39	0.25	4.031	2.453–6.625	1.63	0.30	0.14	****	ab
F_2_	0.99	0.31	2.681	1.906–3.773	0.77	0.24	0.07	****	b
Es_LL*Cross type									****
W	1.68	0.26	5.377	3.797–7.616	1.71	0.27	0.13	****	a
BC_w_	1.70	0.26	5.470	3.894–7.683	1.61	0.25	0.14	****	a
F_1_	1.23	0.20	3.407	2.318–5.007	1.18	0.19	0.088	****	ab
F_2_	0.91	0.26	2.484	1.754–3.519	0.76	0.22	0.061	****	b
Emergdate*Cross type									****
W	−0.155	0.025	0.857	0.828–0.887	−0.024	0.0038	−0.003	****	a
BC_w_	−0.132	0.025	0.876	0.846–0.907	−0.019	0.0036	−0.003	****	a
F_1_	−0.069	0.018	0.933	0.902–0.966	−0.010	0.0024	−0.001	****	b
F_2_	−0.054	0.025	0.948	0.915–0.982	−0.008	0.0035	−0.001	**	b

Selection differentials (*s*) with their standard errors (SE); odds ratios (Odds) and their 95% confidence intervals (95% CI); standardized (*s’*) and average selection differentials (*s*-*avgdif*) for early season height (Es_Ht), early season leaf length (Es_LL), and emergence date (EmergDate). SE(*s’*) are the standard errors for the standardized selection differentials. Significance (Signif.) for a trait's selection differential—trait experienced changes in phenotype due to selection (direct and indirect). Trait*Cross type showing significance for regression separation (RS) had significant differences among cross types for that trait's selection differential; different letters among cross types—significantly different at *P* < 0.05. Significance: **P < 0.01, ****P < 0.0001.

**Table 4:** Cross type specific selection gradients for early season height (Es_Ht), early season leaf length (Es_LL), and emergence date (EmergDate)

Selection Gradients—Cross type									
	*β*	SE	Odds	95% CI	*β*‘	SE (*β*‘)	*β*-*avggrad*	Signif.	RS
Es_Ht*Cross type									ns
W	0.026	0.46	1.026	0.563–1.871	0.020	0.36	0.0014	ns	a
BC_w_	0.029	0.45	1.030	0.580–1.826	0.024	0.37	0.0021	ns	a
F_1_	0.19	0.34	1.211	0.625–2.346	0.16	0.29	0.012	ns	a
F_2_	0.31	0.42	1.363	0.830–2.237	0.40	0.54	0.032	ns	a
Es_LL*Cross type									**
W	1.76	0.40	5.790	3.447–9.728	1.73	0.39	0.12	****	a
BC_w_	1.66	0.39	5.253	3.200–8.624	1.75	0.41	0.15	****	a
F_1_	0.87	0.30	2.382	1.326–4.280	0.90	0.31	0.066	**	ab
F_2_	0.66	0.40	1.935	1.140–3.284	0.79	0.48	0.063	*	b
Emergdate*Cross type									ns
W	0.015	0.050	1.015	0.951–1.084	0.10	0.32	0.0070	ns	a
BC_w_	−0.006	0.049	0.994	0.934–1.059	−0.039	0.34	−0.0034	ns	a
F_1_	−0.053	0.037	0.948	0.882–1.020	−0.38	0.27	−0.028	ns	a
F_2_	−0.007	0.050	0.993	0.930–1.060	−0.052	0.36	−0.0041	ns	a

Selection gradients (*β*) with their standard errors (SE); odds ratios (Odds) and their 95% confidence intervals (95% CI); standardized (*β*’) and average selection gradients (*β* –*avggrad*) for early season height (Es_Ht), early season leaf length (Es_LL), and emergence date (EmergDate). SE (*β*’) are the standard errors for the standardized selection gradients. Significance (Signif.) for a trait's selection gradient—trait was a direct target of natural selection. Trait*Cross type showing significance for regression separation (RS) had significant differences among cross types for that trait's selection gradient; different letters among cross types—significantly different at *P* < 0.05. Significance: *P < 0.05, **P < 0.01, **** *P* < 0.0001, ns—not significant, 0.05.

#### Selection by density

Importantly, effects of competitive treatments could have affected the results of our cross type specific selection analyses. Biased relationships between traits and fitness can be created if environment is correlated with traits of interest and fitness (Rausher 1992). Thus, we performed density-specific selection analysis as it was the only environmental treatment that influenced both early season traits and survival to anthesis (Tables S2–S4). Total phenotypic selection (*s*) on early season traits was greatest in higher density, but density did not have a significant influence on the intensities of direct selection (*β*), suggesting that our cross type specific selection analysis had not been influenced by the environmental treatments imposed during the experiment (Table S6). Therefore, we will not discuss further the influence that our competitive treatments (i.e., density) may have had on our cross type selection analysis.

## Discussion

Studies of ecological dynamics and natural selection in crop–wild hybrid zones are essential to gain a mechanistic understanding of the process of plant hybridization and introgression at the interface between agricultural and unmanaged landscapes. Our study using sunflowers is unique in terms of the combination of: (i) using multiple crop–wild hybrid cross types grown together with their wild counterparts, (ii) performing overall and cross typespecific phenotypic selection analysis on multiple early season traits in a hybrid zone setting, and importantly, (iii) being conducted in a realistic field setting. As seeds of all four cross types overwintered, germinated in the spring, and many individuals from each cross type went on to flower the following summer, all cross types should be able to shepherd crop alleles through the process of introgression following sunflower crop–wild hybridization. Both the percent crop alleles that a cross type possessed and the identity of the maternal parent heavily influenced measured traits. Although selection differentials (*s*) showed that there was selection for earlier spring emergence and larger early season plant size, selection gradients (*β*) demonstrated that direct selection only occurred to increase early season leaf length. This finding suggests that crop alleles contributing to favored early season trait values and linked regions are likely to introgress into wild sunflower populations following crop–wild hybridization both through direct and indirect selection, depending on the trait. We also found that the wild and BC_w_ cross types experienced greater intensities of direct selection on increased early season leaf length as compared to the F_2_ cross type, which may have led to the similar, albeit not direct, cross type selection patterns for earlier spring emergence and greater early season height. These findings suggest that the introgression of crop alleles underlying both earlier spring emergence and greater early season plant size may be more likely to occur through the BC_w_ cross type route.

### Overwintering and spring emergence

BC_w_, F_1_, and F_2_ sunflower crop–wild hybrids can all begin their life cycles as emerged seedlings in crop–wild hybrid zones. All cross types overwintered and had spring seedling emergence rates of at least 41% (Fig.1). Two clear patterns were observed. First, wild-produced cross types (W, BC_w_, and F_1_) had higher emergence rates than the cross type produced on F_1_-maternal plants (F_2_). This may have been due to increased fall emergence (i.e., lack of dormancy) and overwintering mortality, possibly due to more permeable seed coverings or greater sensitivity of unemerged, but germinated, seedlings of the F_2_ cross type (Weiss et al. 2013; Pace et al. 2015). Second, emergence increased for the wild-produced cross types as percent crop alleles decreased. We expected higher emergence rates in crop–wild hybrids compared to their wild counterpart due to reports of higher spring germination rates in F_1_ sunflower crop–wild hybrids (Snow et al. 1998; Mercer et al. 2006b). However, recent germination and emergence studies report trends in line with our findings (Alexander et al. 2014; Pace et al. 2015). Importantly, crop–wild hybrid cross types also closely resembled wilds in average seedling emergence date and shape of emergence curves (Figs1 and 2). Crop–wild hybrid seeds exhibiting germination behavior similar to their wild counterparts may be more likely to persist and further reproduce with wild plants in a crop–wild hybrid population (Ross and Harper 1972; Rees and Long 1992; Adler et al. 1993). Thus, hybrid sunflower cross types should persist in wild populations, compete with their wild counterparts for resources (Rees and Long 1992), and contribute pollen and seed to subsequent generations and, therefore, should not provide a barrier to the introgression of crop traits or particular alleles into wild populations (Landbo and Jørgensen 1997; Mercer et al. 2006b).

### Vegetative growth and survival to anthesis

Sunflower crop–wild hybrids were similar in size or larger than their wild counterparts in both leaf length and height throughout their vegetative growth. The F_2_ cross type was consistently the largest both in early season height and leaf length (Figs3A–D) probably because F_2_ seeds were the largest due to being produced on F_1_ maternal plants (Westoby et al. 1992; Leishman et al. 2000; Weiss et al. 2013). The increased size of F_2_ seeds likely means they store more nutrients (Leishman et al. 2000), exhibit faster root growth (Wulff 1986), and compete more intensively during vegetative growth (Geritz et al. 1999; Rees et al. 2001). Observed differences between the F_1_ and F_2_ cross types could have also been influenced by a number of other processes (i.e., epistasis, recombination, uncovering of recessive alleles, and overdominance; Rieseberg et al. 1999). Nevertheless, regardless of the underlying cause, the possible competitive superiority of the F_2_ cross type could negatively influence the reproductive output of, and possibly selection on, neighboring wild-produced cross types (Weiner 1985, 1990).

As most individuals from each cross type that emerged also survived to anthesis in high numbers (>84%; Fig.4), the vegetative portion of the life cycle does not provide a strong barrier to the introgression of cultivated alleles into wild populations. Competition during the vegetative portion of the life cycle did not cause excessive mortality of any cross type. Survival to anthesis of a cross type would need to be zero to form a barrier to introgression, so introgression can proceed via sib-crossing of F_1_'s and/or backcrossing of F_1_'s onto their wild counterparts (Anderson and Hubricht 1938; Heiser 1973). Nevertheless, some cross types appear to better weather competition and may have differential abilities to branch and produce seed heads, thereby resulting in differential seed production (Mercer et al. 2006b; Mercer et al. 2014).

We observed a decrease in the probability of flowering in the first year as the percent crop alleles increased or if seeds were produced on an F_1_ maternal parent. Nonetheless, all hybrid cross types had a survival rate between 34% and 54% (Fig.4). As was the case for spring emergence (Fig.1), cross type differences may have been due to untimely fall germination, seed or seedling mortality during the winter and early spring, or continued seed dormancy; the first two might be most likely for our F_1_-produced cross type and the latter—for the wild-produced cross types (Pace et al. 2015). Thus, germination/dormancy related traits had a large influence on the percentage of each cross type that flowered in the first year. The inability of the F_2_ cross type to contribute dormant seed to the seed bank (Pace et al. 2015) means F_2_ plants likely only spatially, and not temporally, contributes to introgression via pollen and seed movement (Linder and Schmitt 1994). This apparent lack of F_2_ seed in the seed bank, in combination with its reduction in spring emergence, suggests it may play less of a role in the process of introgression than the BC_w_ cross type. However, sib mating followed by backcrossing may enhance the likelihood of introgression (Wall 1970), suggesting that F_2_ crop–wild hybrids may still play an important role in the introgression process.

### Hybrid zone evolution: likelihood of crop allele introgression

Phenotypic selection analysis elucidates natural selection and, therefore, can provide insight into traits likely to introgress into wild populations following crop–wild hybridization. Fitness increased with earlier seedling emergence, greater early season height, and greater early season leaf length (Table1), but the only trait included in the analysis that experienced direct selection (*β*) was early season leaf length (Table2). As a result, unless influenced by traits not included in the analysis, selection for increased early season leaf length may have dictated selection for earlier emergence and increased early season height (Lande and Arnold 1983). Importantly, these findings suggest that crop alleles shifting these three trait values toward those favored by natural selection may readily introgress into wild sunflower populations unless linked to other crop alleles selected against in wild environments (Linder et al. 1998; Dechaine et al. 2009). Additionally, the wild cross type may not evolve to the optimal phenotype favored by natural selection because of genetic correlations (Etterson and Shaw 2001). If so, recombination during hybridization may break up these genetic correlations, allowing crop alleles underlying favored trait values a greater likelihood of introgressing. Given the ecological importance of spring emergence timing and seedling size, wild plants that emerge earlier and have larger seedlings (from whatever source, e.g., crop alleles or other) will be expected to have greater survival, so that could augment wild fitness as a whole.

Our phenotypic selection findings generally agree with previous studies conducted in sunflower (Baack et al. 2008; Dechaine et al. 2009; Mercer et al. 2011) although discrepancies among studies indicate natural selection can vary spatially and temporally (Kingsolver et al. 2012). For instance, Baack et al. (2008) and Dechaine et al. (2009) did not identify direct selection on increased leaf size in Nebraska, which may be due to environment-specific selective pressures such as variation in herbivory. Other studies found evidence of direct selection operating on increased height (Dechaine et al. 2009; Mercer et al. 2011), contrary to our findings (Table2). The importance of height may have been reduced in our analysis because our trait measurements were taken substantially earlier in the life cycle than in the previously mentioned studies. If this is indeed the case, then it could point to temporal variation in patterns of natural selection—the intensity of natural selection occurring on a given trait can vary throughout the growing season.

For introgression of cultivated alleles to occur, various cross types need to transfer these alleles into subsequent generations. In other words, cross types need to provide routes for cultivated allele introgression. In hybrid zones, cross types experiencing greater selection intensities for crop-like trait values may be more likely than others to successfully act as routes for the introgression of crop alleles underlying these traits. In our study, direct selection for increased early season leaf length varied by cross type indicating natural selection occurred on the cross types differently (Table4). Selection gradients (*β*) for increased early season leaf length were more intense for the wild and BC_w_ cross types and less intense for the F_2_ cross types—the F_1_ cross type did not significantly differ from either of these two groups (Table4). As the formation of F_1_ crop–wild hybrids is a prerequisite for introgression, a selection gradient (*β*) of zero for this cross type would indicate a barrier to the process of introgression (although introgression could still occur through neutral processes). For the F_1_ cross type, our observed selection gradient (*β*) of 0.87 for early season leaf length is sufficient to invoke ‘rapid microevolutionary changes’ (Kingsolver et al. 2012; pp. 3), assuming moderate trait heritability (Falconer 1981). Thus, the F_1_ cross type does not provide a barrier to the introgression of crop alleles contributing to an increase in early season leaf length.

The introgression into wild sunflower populations of cultivated alleles underlying increased early season leaf length may occur via both the BC_w_ and F_2_ routes. For BC_w_ plants to be produced, F_1_ plants must survive to anthesis, produce viable pollen, and overlap in flowering time with wild sunflower populations; all of which have been shown to occur (Fig.4; Snow et al. 1998; Terzić et al. 2006). As direct selection was nearly twice as intense in the BC_w_ cross type as in the F_1_ cross type (Table4), cultivated alleles underlying increased early season leaf length are likely to introgress through the BC_w_ route after crop–wild hybridization. The F_2_ cross type had the greatest early season leaf length of all cross types (Fig.3D), likely due to being produced on F_1_ maternal plants, which produce larger seeds (Weiss et al. 2013), yet it also experienced the least intense direct selection for increased early season leaf length (Table4). Nonetheless, the F_2_ cross type exhibited a selection gradient (*β*) of 0.66 for increased early season leaf length, which suggests a strong likelihood of introgression of cultivated alleles conferring greater early season leaf length via the F_2_ route. As natural selection intensities guide the introgression process, the reduced selection intensity of the F_2_ cross type when compared to that of the BC_w_ may suggest that selection favors introgression via the BC_w_ route (Table4).

### Potential for wild sunflower genetic diversity loss

In addition to the mechanistic understanding of the introgression process gleaned from this study, our findings may provide insight into the likelihood of genetic diversity loss in wild sunflower populations following crop toward wild hybridization and subsequent introgression. Our data generally suggest that genetic diversity loss via demographic swamping is unlikely in wild sunflower populations following hybridization as we did not note a reduction in hybrid fitness (i.e., survival to flowering) when compared to that of the parental populations (Wolf et al. 2002). However, seed production can be lower among crop–wild hybrid sunflower cross types than wild plants, although differences diminish under more competitive conditions (Mercer et al. 2014). Thus, predicting the occurrence of demographic swamping is not straight forward and conclusions will likely hinge on both the fitness measure used and the hybrid zone environment.

Loss of genetic diversity via genetic assimilation seems more likely. While originally increasing diversity due to the addition of novel alleles, the introgression of crop-like traits and their underlying alleles into wild populations can eventually lead to the replacement of wild alleles in the genes under selection and linked loci. This genetic diversity loss is referred to as genetic assimilation (Ellstrand 1992). As natural selection in crop–wild hybrid zones ultimately dictates crop toward wild introgression, our overall selection analyses results—selection favoring earlier spring emergence and greater seedling size (Tables1 and 2), both crop-like traits—suggest that wild sunflower genes encoding spring emergence and seedling size, and linked genetic loci, are vulnerable to genetic assimilation. We might expect that more frequent gene flow events and stronger selection coefficients may increase the risk of this outcome (Ellstrand 2003b). The overall selection coefficients in our experimental hybrid zone (Tables1 and 2) were likely more than sufficient to promote introgression and subsequent genetic assimilation as selection coefficients as low as 0.15 can lead to evolutionary change (Kingsolver and Pfennig 2007). However, as selection coefficients can vary by year, it would take persistent selection for crop alleles to see this affect—something a single year of study cannot completely elucidate. The applied relevance of the insight derived here is that reduction in genetic diversity in plant populations can reduce adaptive potential, with implications for population persistence and ecosystem function (Jump and Peñuelas 2005; Jump et al. 2009).

### Additional studies to further our understanding of introgression

This type of work helps us to predict the potential for introgression of crop alleles associated with particular traits, provides insight into the phenotypes and fitness of multiple generations of hybrids found in natural crop–wild hybrid zones, and may illuminate traits potentially vulnerable to diversity loss. Our findings suggest that regions of the genome controlling spring emergence and seedling size are strong candidates for future studies assessing changes in genetic diversity in crop–wild hybrid zones. Nevertheless, predicting the phenotypes and evolutionary dynamics of future generations can be challenging for a number of reasons. As we saw here in the differences between F1 and F2 cross types, maternal genetic effects, as well as nuclear genetic composition, can influence both plant phenotype and intensities of natural selection (Weiss et al. 2013; Alexander et al. 2014; Pace et al. 2015). This variation among generations from a single hybrid zone affects how introgression proceeds and will affect the phenotypes of future generations, partly through maternal and partly through nuclear genetic effects (Alexander et al. 2014). Moreover, the genetic background of the hybridizing wild and crop populations themselves can influence the phenotypes of hybrid generations and possibly selection dynamics (Mercer et al. 2006b). Utilizing further advanced generation hybrid cross types and populations from additional locations should help elucidate the influence that maternal and genetic backgrounds have on the introgression process.

Additional phenotypic selection methods may have provided an even deeper understanding of crop toward wild introgression dynamics. Survival is the most commonly used fitness measure in selection studies (Kingsolver and Diamond 2011) and has been demonstrated to be an appropriate fitness measure when assessing selection on plant size (Kingsolver and Pfennig 2007) and seedling emergence (Verdú and Traveset 2005; Mercer et al. 2011). Yet as evolutionary change is caused by the cumulative effect of survival, mating success, fecundity, and so on (Siepielski et al. 2011), studying multiple fitness components, and ultimately performing selection analyses on total fitness (e.g., Shaw et al. 2008), would increase our understanding of natural selection in hybrid zones. Our use of survival to flowering captured a particular snapshot of selection, which can differ in intensity and direction when different fitness measures are used (Kalisz 1986; but see Kingsolver and Diamond 2011 for selection on size). Similarly, testing for stabilizing and disruptive selection may provide additional insight as the former could ultimately influence intensities of directional selection on study traits.

We have shown that selection directly favors early season leaf length in a sunflower crop–wild hybrid zone, but we have not identified the selective pressure(s) responsible. Neither have we shown that the trait has a causal relation with fitness. A logical next step is to identify the selective pressure(s) responsible for the observed selection patterns. Information gleaned from phenotypic selection studies can be used to develop hypotheses driven studies where selection pressure intensities are experimentally altered (Wade and Kalisz 1990). A change in the selection gradient intensity of a trait when causal selective pressure intensities are altered indicates a relationship between the agent (selective pressure) and target (trait) of selection. Additionally, studying the interaction between selection gradient and selection pressure intensities provides insight into causal relationships between traits and fitness as the interaction between the former ‘causes fitness’ (Wade and Kalisz 1990). Given that differential selection was observed among our cross types and the cross types were interacting, an interesting question that arises is, How do crop–wild hybrid cross types inhabiting a crop–wild hybrid zone influence selection on each other? For instance, Did the large F_2_ cross type shape the selection intensities experienced by the wild-produced cross types? Studies comparing cross type selection patterns in experimental crop–wild hybrid zones with and without certain cross type(s) provide a way to identify whether certain cross types are the causative selective pressure(s) responsible for observed selection patterns. Integrating this type of analysis into crop–wild hybrid introgression studies will provide a more full view of the ecological processes influencing introgression by providing insight into hybrid zone competition dynamics.

## Conclusion

Spring seedling emergence characteristics and survival to anthesis do not provide strong barriers to the introgression of cultivated sunflower alleles into wild sunflower populations. Overall phenotypic selection analysis indicates direct selection for greater early season leaf size could promote the introgression of crop alleles contributing to this trait as well as those for earlier emergence and greater early season height. There were multiple cross types that provide likely pathways to the introgression of the genetic architecture underpinning earlier seedling emergence and greater seedling size. The introgression of beneficial alleles from the crop for these traits could exacerbate genetic diversity loss in wild sunflower populations. Genetic regions underlying these traits are good candidates for studies focusing on genetic diversity loss in wild sunflower populations following crop toward wild introgression. Additional issues that remain to be addressed include: (i) To what degree do other advanced generations hybrids, including further backcrosses and sib-crosses, facilitate introgression; (ii) how different are the barriers, or lack thereof, for different traits or suites of traits; and (iii) what factors influence the likelihood that wild *H. annuus* could act as a bridge for crop alleles to introgress into other species in the genus.

## Data archiving statement

Data available from the Dryad Digital Repository: http://dx.doi.org/10.5061/dryad.p0kj9.

## Supporting Information

**Table S1.** Pairwise Pearson correlation coefficients among all trait values monitored during our three census periods. **Table S2.**anova table for height during all three census periods. **Table S3.**anova table for leaf length during all three censuses. **Table S4.**anova table for survival to anthesis (emerged) and probability that a planted seed flowered in the first year (sowed). **Table S5.**anova tables for % spring emergence and average emergence day. **Table S6.** Density specific selection differentials and gradients for early season height (Es_Ht), early season leaf length (Es_LL), and emergence date (EmergDate).
