# Supplementary material for: Life history traits and phenotypic selection among sunflower crop–wild hybrids and their wild counterpart: implications for crop allele introgression
Source: Evol Appl. 2015 May 12;8(5):510–24. doi: 10.1111/eva.12261 (PMC4430773; doi:10.1111/eva.12261)
Supplement: Supplementary file 1 — Table S1. Pairwise Pearson correlation coefficients among all trait values monitored during our three census periods. Table S2.anova table for height during all three census periods. Table S3.anova table for leaf length during all three censuses. Table S4.anova table for survival to anthesis (emerged) and probability that a planted seed flowered in the first year (sowed). Table S5.anova tables for % spring emergence and average emergence day. Table S6. Density specific selection differentials and gradients for early season height (Es_Ht), early season leaf length (Es_LL), and emergence date (EmergDate). [file eva0008-0510-sd1.docx]

**Supplementary materials**

Table S1. Pairwise Pearson correlation coefficients among all trait values monitored during our three census periods

|  | EmergeDate | C1_LL | C2_LL | C3_LL | C1_WL | C2_WL | C3_WL | C1_Ht | C2_Ht | C3_Ht | C2_Node | C3_Node | C2_Lvs | C3_Lvs |
| --- | --- | --- | --- | --- | --- | --- | --- | --- | --- | --- | --- | --- | --- | --- |
| EmergeDate | 1 | -0.6125 | -0.5405 | -0.4766 | -0.5143 | -0.5085 | -0.4562 | -0.5429 | -0.5085 | -0.5282 | -0.5856 | -0.4974 | -0.5842 | -0.4495 |
| C1_LL | <0.0001 | 1 | 0.8445 | 0.7251 | 0.8513 | 0.8205 | 0.7283 | 0.7364 | 0.6885 | 0.0.6867 | 0.7226 | 0.72853 | 0.71126 | 0.6556 |
| C2_LL | <0.0001 | <0.0001 | 1 | 0.8781 | 0.7357 | 0.9002 | 0.8645 | 0.6506 | 0.6751 | 0.7333 | 0.7628 | 0.8132 | 0.7630 | 0.7515 |
| C3_LL | <0.0001 | <0.0001 | <0.0001 | 1 | 0.6242 | 0.8193 | 0.9168 | 0.5288 | 0.5476 | 0.6961 | 0.6896 | 0.7941 | 0.6897 | 0.7469 |
| C1_WL | <0.0001 | <0.0001 | <0.0001 | <0.0001 | 1 | 0.8349 | 0.6885 | 0.7589 | 0.6264 | 0.5863 | 0.6930 | 0.6831 | 0.6652 | 0.6273 |
| C2_WL | <0.0001 | <0.0001 | <0.0001 | <0.0001 | <0.0001 | 1 | 0.8793 | 0.6970 | 0.6509 | 0.6570 | 0.7676 | 0.8040 | 0.7506 | 0.7407 |
| C3_WL | <0.0001 | <0.0001 | <0.0001 | <0.0001 | <0.0001 | <0.0001 | 1 | 0.5586 | 0.5314 | 0.6347 | 0.7060 | 0.8095 | 0.6979 | 0.7608 |
| C1_Ht | <0.0001 | <0.0001 | <0.0001 | <0.0001 | <0.0001 | <0.0001 | <0.0001 | 1 | 0.7500 | 0.5530 | 0.6338 | 0.5884 | 0.6156 | 0.52338 |
| C2_Ht | <0.0001 | <0.0001 | <0.0001 | <0.0001 | <0.0001 | <0.0001 | <0.0001 | <0.0001 | 1 | 0.6749 | 0.6494 | 0.6016 | 0.6360 | 0.5262 |
| C3_Ht | <0.0001 | <0.0001 | <0.0001 | <0.0001 | <0.0001 | <0.0001 | <0.0001 | <0.0001 | <0.0001 | 1 | 0.6185 | 0.7014 | 0.6233 | 0.6674 |
| C2_Node | <0.0001 | <0.0001 | <0.0001 | <0.0001 | <0.0001 | <0.0001 | <0.0001 | <0.0001 | <0.0001 | <0.0001 | 1 | 0.7608 | 0.9550 | 0.6644 |
| C3_Node | <0.0001 | <0.0001 | <0.0001 | <0.0001 | <0.0001 | <0.0001 | <0.0001 | <0.0001 | <0.0001 | <0.0001 | <0.0001 | 1 | 0.7480 | 0.8501 |
| C2_Lvs | <0.0001 | <0.0001 | <0.0001 | <0.0001 | <0.0001 | <0.0001 | <0.0001 | <0.0001 | <0.0001 | <0.0001 | <0.0001 | <0.0001 | 1 | 0.6745 |
| C3_Lvs | <0.0001 | <0.0001 | <0.0001 | <0.0001 | <0.0001 | <0.0001 | <0.0001 | <0.0001 | <0.0001 | <0.0001 | <0.0001 | <0.0001 | <0.0001 | 1 |

Note: All pairwise Pearson correlation coefficients were calculated to identify a moderately independent set of traits. Traits include emergence date (EmergDate), leaf length (LL), leaf width (WL), height (Ht), number of nodes (Node), and number of leaves (Lvs). Trait data was collected during three census periods (C1, C2 and C3) from our experimental site in Jefferson County, Kansas – see text for details on collection dates. Below the diagonal line contains p-values for each pairwise comparison and above the diagonal contains Pearson correlation coefficients. The darker the correlation coefficient box, the greater the correlation. Moderate Pearson correlation coefficients are represented by the lightest shade of grey (±0.40-0.60), while the remaining correlations are darker shades of grey and are either highly correlated (0.6-0.8) or very highly correlated (0.8-1.0). The three traits used in our phenotypic selection analysis fall into three differentiated groups that sometimes obtain very high levels of correlation within, but only moderate to high levels of correlation between groups.

Table S2. ANOVA table for height during all three census periods

|  | Census 1 Height | | | Census 2 Height | | | Census 3 Height | | |
| --- | --- | --- | --- | --- | --- | --- | --- | --- | --- |
| Source of variation | df^a^ | F | P | df^a^ | F | P | df^a^ | F | P |
| Density | 2,55 | 4.91 | 0.011* | 2,55 | 2.36 | 0.1037 | 2,55 | 37.69 | <0.0001* |
| Hybrid percent | 1,55 | 0.19 | 0.6618 | 1,55 | 0.02 | 0.8875 | 1,55 | 0.09 | 0.7705 |
| Vegetation | 1,55 | 0.46 | 0.5018 | 1,55 | 2.62 | 0.1115 | 1,55 | 26.87 | <0.0001* |
| Cross type | 3,180 | 242.10 | <0.0001* | 3,179 | 97.30 | <0.0001* | 3,180 | 28.07 | <0.0001* |
| Hyb*Veg | 1,55 | 0.21 | 0.6487 | 1,55 | 0.41 | 0.5254 | 1,55 | 0.16 | 0.6944 |
| Hyb*Dens | 2,55 | 2.89 | 0.0641 | 2,55 | 2.81 | 0.0688 | 2,55 | 0.37 | 0.6935 |
| Hyb*Cross | 3,180 | 0.17 | 0.9165 | 3,179 | 1.89 | 0.1337 | 3,180 | 0.59 | 0.6234 |
| Veg*Dens | 2,55 | 2.42 | 0.098 | 2,55 | 0.15 | 0.8633 | 2,55 | 1.57 | 0.2179 |
| Veg*Cross | 3,180 | 0.86 | 0.4641 | 3,179 | 1.48 | 0.2226 | 3,180 | 0.55 | 0.6477 |
| Dens*Cross | 6,180 | 0.30 | 0.9371 | 6,179 | 0.87 | 0.5215 | 6,180 | 1.00 | 0.424 |
| Hyb*Veg*Dens | 2,55 | 0.25 | 0.7826 | 2,55 | 0.17 | 0.8447 | 2,55 | 0.37 | 0.6931 |
| Hyb*Veg*Cross | 3,180 | 0.64 | 0.5878 | 3,179 | 0.05 | 0.985 | 3,180 | 0.80 | 0.4978 |
| Hyb*Dens*Cross | 6,180 | 1.11 | 0.3563 | 6,179 | 1.47 | 0.1902 | 6,180 | 0.49 | 0.8157 |
| Veg*Dens*Cross | 6,180 | 1.58 | 0.1549 | 6,179 | 1.43 | 0.2071 | 6,180 | 0.65 | 0.6934 |
| Hyb*Veg*Dens*Cross | 6,180 | 1.88 | 0.0872 | 6,179 | 1.89 | 0.0843 | 6,180 | 1.15 | 0.3377 |

^a^ Numerator df followed by denominator df

Note: ANOVA table includes degrees of freedom, F-values and P-values for height during our three censuses. All fixed effects and factorial combinations of fixed effects that were employed during our field experiment in Jefferson County, Kansas, are included. Significant effects (<0.05) are represented by an *.

Table S3. ANOVA table for leaf length during all three censuses

|  | Census 1 Leaf Length | | | Census 2 Leaf Length | | | Census 3 Leaf Length | | |
| --- | --- | --- | --- | --- | --- | --- | --- | --- | --- |
| Source of variation | df^a^ | F | P | df^a^ | F | P | df^a^ | F | P |
| Density | 2,55 | 15.5 | <0.0001* | 2,55 | 92.50 | <0.0001* | 2,55 | 141.30 | <0.0001* |
| Hybrid percent | 1,55 | 1.38 | 0.2459 | 1,55 | 0.31 | 0.5783 | 1,55 | 0.75 | 0.3896 |
| Vegetation | 1,55 | 3.41 | 0.0703 | 1,55 | 17.30 | 0.001* | 1,55 | 24.41 | <0.0001* |
| Generation | 3,180 | 97.28 | <0.0001* | 3,179 | 71.90 | <0.0001* | 3,180 | 21.26 | <0.0001* |
| Hyb*Veg | 1,55 | 0.08 | 0.7743 | 1,55 | 0.08 | 0.7748 | 1,55 | 0.36 | 0.5532 |
| Hyb*Dens | 2,55 | 1.31 | 0.2772 | 2,55 | 0.80 | 0.4553 | 2,55 | 0.00 | 1.0000 |
| Hyb*Cross | 3,180 | 0.19 | 0.9051 | 3,179 | 0.32 | 0.8078 | 3,180 | 0.24 | 0.8711 |
| Veg*Dens | 2,55 | 2.09 | 0.1332 | 2,55 | 5.57 | 0.0062* | 2,55 | 4.48 | 0.0157* |
| Veg*Cross | 3,180 | 0.52 | 0.6703 | 3,179 | 1.02 | 0.3834 | 3,180 | 0.88 | 0.4548 |
| Dens*Cross | 6,180 | 1.09 | 0.3726 | 6,179 | 0.89 | 0.5054 | 6,180 | 0.90 | 0.4978 |
| Hyb*Veg*Dens | 2,55 | 0.22 | 0.8016 | 2,55 | 0.66 | 0.5231 | 2,55 | 1.87 | 0.1643 |
| Hyb*Veg*Cross | 3,180 | 0.36 | 0.7853 | 3,179 | 0.24 | 0.8693 | 3,180 | 0.10 | 0.9625 |
| Hyb*Dens*Cross | 6,180 | 1.48 | 0.1875 | 6,179 | 0.88 | 0.5089 | 6,180 | 0.47 | 0.8277 |
| Veg*Dens*Cross | 6,180 | 1.19 | 0.3152 | 6,179 | 0.96 | 0.4519 | 6,180 | 1.01 | 0.4232 |
| Hyb*Veg*Dens*Cross | 6,180 | 1.53 | 0.1709 | 6,179 | 1.13 | 0.3474 | 6,180 | 1.73 | 0.1171 |

^a^ Numerator df followed by denominator df

Note: ANOVA table includes degrees of freedom, F-values and P-values for leaf length during or three censuses. All fixed effects and factorial combinations of fixed effects that were employed during our field experiment in Jefferson County, Kansas, are included. Significant effects (<0.05) are represented by an *.

Table S4. ANOVA table for survival to anthesis (emerged) and probability that a planted seed flowered in the first year (sowed)

| Survival to anthesis | Emerged | | | Sowed | | |
| --- | --- | --- | --- | --- | --- | --- |
| Source of variation | df^a^ | F | P | df^a^ | F | P |
| Density | 2,55 | 30.05 | <0.0001* | 2,55 | 7.63 | 0.0012* |
| Hybrid percent | 1,55 | 0.16 | 0.6932 | 1,55 | 0.34 | 0.5633 |
| Vegetation | 1,55 | 0.34 | 0.5645 | 1,55 | 0.02 | 0.8785 |
| Generation | 3,186 | 1.39 | 0.2462 | 3,180 | 47.44 | <0.0001* |
| Hyb*Veg | 1,55 | 0.5 | 0.4812 | 1,55 | 0.09 | 0.7686 |
| Hyb*Dens | 2,55 | 0.36 | 0.6999 | 2,55 | 1.49 | 0.2351 |
| Hyb*Cross | 3,186 | 0.76 | 0.5184 | 3,180 | 0.49 | 0.6893 |
| Veg*Dens | 2,55 | 0.1 | 0.9065 | 2,55 | 0.01 | 0.9925 |
| Veg*Cross | 3,186 | 0.7 | 0.5535 | 3,180 | 1.11 | 0.3458 |
| Dens*Cross | 6,186 | 1.43 | 0.2039 | 6,180 | 1.05 | 0.3962 |
| Hyb*Veg*Dens | 2,55 | 1.1 | 0.3389 | 2,55 | 0.07 | 0.9355 |
| Hyb*Veg*Cross | 3,186 | 0.62 | 0.603 | 3,180 | 0.41 | 0.7461 |
| Hyb*Dens*Cross | 6,186 | 0.38 | 0.8908 | 6,180 | 0.42 | 0.8631 |
| Veg*Dens*Cross | 6,186 | 0.43 | 0.8617 | 6,180 | 0.48 | 0.8237 |
| Hyb*Veg*Dens*Cross | - | - | - | 6,180 | 0.81 | 0.5631 |

^a^ Numerator df followed by denominator df

Note: ANOVA table includes degrees of freedom, F-values and P-values for survival to anthesis and probability that a planted seed flowered in the first year. Survival to anthesis was calculated from seeds that emerged in the spring while probability that a planted seed flowered in the first year was calculated from seeds sowed in the fall. All fixed effects and factorial combinations of fixed effects that were employed during our field experiment in Jefferson County, Kansas, are included. Significant effects (<0.05) are represented by an *.

Table S5. ANOVA tables for % spring emergence and average emergence day

|  | % Spring emergence | | | Average emergence day | | |
| --- | --- | --- | --- | --- | --- | --- |
| Source of variation | df^a^ | F | P | df^a^ | F | P |
| Density | 2,55 | 0.04 | 0.9595 | 2,55 | 0.25 | 0.7782 |
| % Hybrid | 1,55 | 0.18 | 0.6725 | 1,55 | 3.90 | 0.0534 |
| Vegetation | 1,55 | 0.01 | 0.9377 | 1,55 | 1.65 | 0.2045 |
| Cross type | 3,180 | 62.38 | <0.0001* | 3,180 | 4.02 | 0.0084* |
| Hyb*Veg | 1,55 | 0.02 | 0.8786 | 1,55 | 1.50 | 0.2254 |
| Hyb*Dens | 2,55 | 1.15 | 0.3255 | 2,55 | 0.44 | 0.6475 |
| Hyb*Cross | 3,180 | 0.19 | 0.9059 | 3,180 | 0.19 | 0.9032 |
| Veg*Dens | 2,55 | 0.05 | 0.9531 | 2,55 | 1.17 | 0.3192 |
| Veg*Cross | 3,180 | 1.24 | 0.2968 | 3,180 | 0.02 | 0.9965 |
| Dens*Cross | 6,180 | 0.58 | 0.7425 | 6,180 | 0.82 | 0.5595 |
| Hyb*Veg*Dens | 2,55 | 0.47 | 0.6286 | 2,55 | 1.05 | 0.3581 |
| Hyb*Veg*Cross | 3,180 | 0.23 | 0.8757 | 3,180 | 0.37 | 0.7778 |
| Hyb*Dens*Cross | 6,180 | 0.57 | 0.7563 | 6,180 | 0.86 | 0.5219 |
| Veg*Dens*Cross | 6,180 | 0.34 | 0.9155 | 6,180 | 0.74 | 0.6209 |
| Hyb*Veg*Dens*Cross | 6,180 | 0.99 | 0.4352 | 6,180 | 1.37 | 0.2302 |

^a^ Numerator df followed by denominator df

Note: ANOVA table includes degrees of freedom, F-values and P-values for % spring emergence and average emergence day. All fixed effects and factorial combinations of fixed effects that were employed during our field experiment in Jefferson County, Kansas, are included. Significant effects (<0.05) are represented by an *.

Table S6. Density specific selection differentials and gradients for early season height (Es_Ht), early season leaf length (Es_LL), and emergence date (EmergDate)

| **Selection Differentials – Density** | | | | | | |  | | |
| --- | --- | --- | --- | --- | --- | --- | --- | --- | --- |
|  | *s* | SE | OR | 95% CI | *s’* | SE( *s’*) | *s*-*avgdif* | Signif. | RS |
| Es_Ht*Density |  |  |  |  |  |  |  |  | * |
| 495 | 1.66 | 0.32 | 5.252 | 3.934-7.012 | 1.61 | 0.31 | 0.30 | **** | a |
| 255 | 1.11 | 0.33 | 3.033 | 2.192-4.195 | 1.06 | 0.31 | 0.086 | **** | b |
| 100 | 1.36 | 0.29 | 3.909 | 2.207-6.924 | 1.29 | 0.27 | 0.044 | **** | ab |
| Es_LL*Density |  |  |  |  |  |  |  |  | **** |
| 495 | 1.85 | 0.20 | 6.337 | 4.782-8.399 | 1.80 | 0.19 | 0.30 | **** | a |
| 255 | 1.13 | 0.14 | 3.088 | 2.333-4.088 | 1.03 | 0.13 | 0.07 | **** | b |
| 100 | 0.92 | 0.24 | 2.508 | 1.691-3.720 | 0.78 | 0.20 | 0.02 | **** | b |
| Emergdate*Density |  |  |  |  |  |  |  |  | **** |
| 495 | -0.144 | 0.026 | 0.866 | 0.842-0.890 | -0.0207 | 0.004 | -0.0060 | **** | a |
| 255 | -0.092 | 0.026 | 0.912 | 0.886-0.939 | -0.0129 | 0.004 | -0.0014 | **** | b |
| 100 | -0.034 | 0.021 | 0.966 | 0.927-1.008 | -0.0032 | 0.003 | -0.0004 | ns | c |
| **Selection Gradients – Density** | | | | | |  |  |  |  |
|  | *Β* | SE | OR | 95% CI | *β* ' | SE (*β* ') | *β* -*avggrad* | Signif. | RS |
| Es_Ht*Density |  |  |  |  |  |  |  |  | ns |
| 495 | 0.161 | 0.279 | 1.174 | 0.790-1.747 | 0.17 | 0.29 | 0.028 | ns | a |
| 255 | -0.14 | 0.208 | 0.869 | 0.578-1.308 | -0.15 | 0.22 | -0.0099 | ns | a |
| 100 | 0.055 | 0.436 | 1.057 | 0.490-2.282 | 0.059 | 0.46 | 0.0016 | ns | a |
| Es_LL*Density |  |  |  |  |  |  |  |  | ns |
| 495 | 1.67 | 0.30 | 5.290 | 3.579-7.818 | 1.71 | 0.30 | 0.29 | **** | a |
| 255 | 1.12 | 0.22 | 3.076 | 1.998-4.735 | 1.23 | 0.24 | 0.083 | **** | a |
| 100 | 0.87 | 0.40 | 2.384 | 1.243-4.573 | 1.03 | 0.47 | 0.028 | ** | a |
| Emergdate*Density |  |  |  |  |  |  |  |  | ns |
| 495 | -0.017 | 0.037 | 0.983 | 0.941-1.027 | -0.12 | 0.26 | -0.020 | ns | a |
| 255 | -0.018 | 0.031 | 0.982 | 0.925-1.042 | -0.13 | 0.22 | -0.0089 | ns | a |
| 100 | -0.004 | 0.053 | 0.996 | 0.913-1.086 | -0.028 | 0.35 | -0.0008 | ns | a |

Note. Selection differentials (*s*) and selection gradients (*β*) accompanied by their standard errors; odds ratios (Odds) along with 95% confidence intervals (95% CI); standardized (*s’* and *β* ') selection coefficients and their standard errors (SE( *s’*)) and (SE (*β* ')), respectively; and average selection coefficients (*s*-*avgdif* and *β* –*avggrad*) for early season height (Es_Ht), early season leaf length (Es_LL), and emergence date (EmergDate). Significance (Signif.) for trait selection differential – trait experienced phenotypic changes due to selection (direct and indirect). Significance (Signif.) for a trait selection gradient – trait was a direct target of natural selection. Trait*Density showing significance for regression separation (RS) exhibited significant differences among cross types selection coefficients for that trait; different letters among crosstypes – significantly different at P < 0.05. Significance: * P < 0.05, ** P < 0.01, *** P < 0.001, **** P < 0.0001, ns – not significant, 0.05.
